# Serum Leptin Levels in Patients with Ocular and Nonocular Behçet's Disease

**DOI:** 10.1155/2007/31986

**Published:** 2007-05-15

**Authors:** F. Nilüfer Yalçındağ, Üçler Kısa, Figen Batıoğlu, Ali Yalçındağ, Özden Özdemir, Osman Çağlayan

**Affiliations:** ^1^Department of Ophthalmology, Faculty of Medicine, Ankara University, 06100 Ankara, Turkey; ^2^Department of Clinical Biochemistry, Faculty of Medicine, Kırıkkale University, 71200 Kırıkkale, Turkey

## Abstract

*Aims*. To investigate serum leptin levels in Behçet's patients with or without ocular involvement compared with healthy subjects and the relationship between serum leptin and uveitis activity in patients with ocular involvement. *Methods*. Fifty-seven patients with Behçet's disease and 20 healthy control subjects were included in this study. While 27 patients had ocular involvement (18 had acute uveitis, 9 had inactive ocular involvement), 30 did not have ocular disease. C-reactive protein, alpha 1-antitrypsin, and serum leptin levels were measured in all samples. *Results*. There was a significant difference between the patients with Behçet's disease and control group for both logarithm of leptin (*P* = .000) and logarithm of CRP (*P* = .031). Logarithm of leptin in non-ocular Behçet's patients was significantly higher compared to its level in ocular Behçet's disease and controls (*P* = .009). There was a significant difference between the patients with active ocular disease and control group (*P* = .03). *Conclusions*. Leptin might have a possible role in the pathogenesis of Behçet's disease.

## 1. INTRODUCTION

Behçet's disease is a chronic, systemic inflammatory disorder affecting multiple organs with a generalized vasculitis [1]. The etiology and pathogenesis of Behçet's disease have not been entirely enlightened. The main immunological features of Behçet's disease consist of increased T- and B-cell responses to heat-shock proteins, increased neutrophil activity, and alterations in cytokine levels, although the interrelationships between and among these features have not been clarified [2]. Serum levels of neutrophil priming cytokines such as TNF, interleukin-1*β*, and IL-8 are determined to be raised in patients with Behçet's disease [3].

Leptin is a peptide hormone that regulates body weight as well as endocrine and immune functions [4–6]. Leptin has structural and functional similarities with members of cytokines family [7]. The leptin receptor is homologous to a subunit of the IL-6-type cytokine receptors [8, 9]. Leptin shares the same signal transduction pathway with cytokines [10].

In light of the fact that leptin might participate in the host response to inflammation, we aimed to investigate changes of serum leptin levels in Behçet's patients with or without ocular involvement compared with healthy subjects and the relationship between serum leptin and uveitis activity in patients with ocular involvement. We compared serum leptin changes with other acute phase reactants, such as CRP or alpha-1 antitrypsin (*α*1-antitrypsin). 

## 2. MATERIAL AND METHODS

Fifty-seven patients with Behçet's disease were included in this study. All patients included in the study met the international diagnostic criteria for Behçet's disease [11]. A complete ophthalmologic examination was performed by ophthalmologists with an interest in Behçet's disease. While 27 patients had ocular involvement, the other 30 did not have ocular disease. All patients with ocular involvement were split into two groups according to the activation of eye involvement to investigate the association between leptin levels and ocular disease activity. Of the 27 patients who had ocular disease, 18 suffered acute uveitis and 9 had inactive ocular involvement at the time of enrollment. None of the Behçet's patients were on systemic steroids or immunosuppressant agents. Twenty healthy control subjects were also included in the study. The subjects who had any systemic or ocular disease were excluded. The subjects were matched for age, sex, and body mass index (BMI). Informed consent was obtained from all participants.

A venous blood sample was taken from each patient and control subject between 8.00 a.m. and 10:00 a.m. after an overnight fast. The blood samples were centrifugated to obtain serum. All serum samples were immediately stored at −80°C until use.

C-reactive protein (CRP), *α*1-antitrypsin, and serum leptin levels were measured in all samples. C-reactive protein and *α*1-antitrypsin levels were measured by the immunoturbidimetric method using commercial kit (Roche Mannheim, Germany) on P 800 autoanalyzer (Roche). Serum samples were assayed for leptin, using enzyme-amplified sensitivity immunoassay kit (Biosource Europe S.A., Nivelles, Belgium).

Serum leptin levels were compared with those measurings of other acute phase reactants, such as CRP or *α*1-antitrypsin.

### 2.1. Statistics

Statistical analysis was performed using SPSS 13.0 software for Windows. As the data were not normally distributed, logarithmic values of CRP (log CRP) and leptin (log leptin) levels were used. Statistical analysis was performed by Student *t* test to compare all Behçet's patients with controls. ANOVA and post hoc test (Tukey HSD) were used to compare ocular Behçet's patients with nonocular Behçet's patients and controls. Kruskal-Wallis and Mann-Whitney *U* tests were used to compare active ocular Behçet's patients with inactive ocular Behçet's patients, nonocular Behçet's patients, and controls.

Pearson's correlation test was used for evaluating the correlation between different parameters in all groups, apart from the group consisting of the patients with inactive eye involvement. Spearman's correlation analysis was used for evaluating that group. Data were presented as mean ± SD. A value of *P* < .05 was considered significant.

## 3. RESULTS

Patient characteristics and laboratory findings are outlined in Table 1. Of the 57 patients in this study, 28 were male (49.1%) and 29 were female (50.9%). There was a significant difference between the patients with Behçet's disease and control group for both log leptin (*P* = .000) and log CRP (*P* = .031). Log leptin in nonocular Behçet's patients was significantly higher compared to its level in ocular Behçet's disease and controls (*P* = .009). Log leptin was not different between patients with ocular and nonocular Behçet's disease. However, there was a significant difference between the patients with active ocular Behçet's disease and control group (*P* = .03).

In patients with Behçet's disease, log leptin was correlated with BMI (*r* = .44, *P* = .001), and *α*1-antitrypsin was correlated with log CRP (*r* = .61, *P* = .000). On the other hand, there was no significant correlation between log leptin and *α*1-antitrypsin or log CRP. In patients with nonocular Behçet's disease, *α*1-antitrypsin was correlated with log CRP (*r* = .56, *P* = .002), and log leptin was correlated with BMI (*r* = .052, *P* = .004). In patients with ocular Behçet's disease, *α*1-antitrypsin was correlated with log CRP (*r* = .73, *P* = .000). In patients with active ocular Behçet's disease, *α*1-antitrypsin was correlated with log CRP (*r* = .79, *P* = .000). In patients with inactive ocular Behçet's disease, log leptin was correlated with BMI (*r* = .86, *P* = .007), and *α*1-antitrypsin was correlated with log CRP (*r* = .81, *P* = .014). In control group, there was no significant correlation between log leptin and *α*1-antitrypsin or BMI and log CRP.

## 4. DISCUSSION

Leptin is primarily produced by adipose tissue. Leptin levels in the systemic circulation are controlled by a variety of factors, especially food intakeand the endocrine system [12]. The innate immune system also participates in the regulation of leptin production.

In experimental animals, leptin levels are acutely increased after administration of the inflammatory stimulus such as lipopolysaccharide (LPS) and turpentine, and proinflammatory cytokines such as tumor necrosis factor-*α* (TNF-*α*) and interleukin-1 (IL-1) [5, 13]. The kinetics of leptinproduction during infectious and inflammatory processes is similar to that of cytokine induction [14].

Data obtained in human studies regarding the regulation of leptin expression in response to infection and inflammation are varied. The administration of IL-1*β* or TNF-*α* results in increased serum leptin levels in healthy subjects [15, 16]. However, no increase in serum leptin levels was found after LPS injection to humans [17]. Leptin concentrations were found to be elevated during an acute inflammatory stimulus such as major surgery [18]. Nevertheless, in the chronic phase of inflammatory diseases such as rheumatoid arthritis and inflammatory bowel disease, inflammation-associated leptin peaks were not observed [19, 20].

Although the etiology and pathogenesis of Behçet's disease are not clear, increased serum levels of inflammatory cytokines have been established to correlate with Behçet's disease activity [3–21]. A correlation between leptin levels and disease activity in patients with Behçet's disease has been reported [22]. Leptin levels in 35 patients with Behçet's disease were also found to be higher than in healthy controls in that study [22]. In another study, 28 males with ocular Behçet's disease were compared with 15 healthy controls, and serum levels of leptin were not significantly different between the groups [23]. In that study, leptin levels were compared between patients with ocular Behçet's disease and healthy subjects; whereas, we compared leptin levels between the groups composed of Behçet's patients with and without ocular involvement and healthy subjects. In our study, patients with Behçet's disease with or without ocular involvement were found to have higher serum leptin and CRP levels compared according to age-, sex-, and BMI-matched controls. However, in Behçet's patients with ocular involvement, serum leptin concentrations were not significantly different from those in Behçet's patients without ocular involvement in our study. The results of our study also demonstrated that the mean serum leptin levels were significantly increased in patients with active uveitis compared to control subjects.

Because serum leptin levels are closely associated with the amount of body fat and differ between males and females, BMI- and sex-matched subjects were enrolled in the groups. The subjects were on systemic steroids or immunosuppressive agents were not included in the study to avoid the possible effects of these agents on the serum leptin levels.

In conclusion, the higher serum leptin levels in patients with Behçet's disease compared to control subjects in the present study might be attributed to a possible role for leptin in the pathogenesis of Behçet's disease. Elevated serum leptin level does not seem to be a risk factor in ocular involvement. However, there might be a correlation between leptin levels and uveitis activity. Future studies would be useful to clarify the potential influence of leptin in Behçet's disease.

## Figures and Tables

**Table 1:** Patient demographics and laboratory findings in patients with ocular and nonocular Behçet's disease and controls.

	Patients with Behçet's disease (BD)					Control subjects
	All BD patients	Nonocular BD	All ocular BD	Active ocular BD	Inactive ocular BD	

Number	57	30	27	18	9	20
Age (years)	33.9 ± 8.1	34.3 ± 8.9	33.6 ± 7.5	33.3 ± 9	34.8 ± 6.6	34.7 ± 8.6
BMI (kg/m^2^)	25.6 ± 4.6	25.9 ± 5.4	25.2 ± 3.5	26 ± 3.4	23.7 ± 3.4	23.8 ± 3.7
Log leptin (ng/mL)	1.9 ± 0.4	2 ± 0.4	1.9 ± 0.4	1.9 ± 0.4	1.9 ± 0.3	1.7 ± 0.1
*α*1-antitrypsin (mg/dL)	180.3 ± 41.7	188.3 ± 66.3	177.6 ± 31.2	180.4 ± 36.8	171.9 ± 15.7	175.9 ± 34.3
Log CRP (mg/L)	1.6 ± 0.6	1.6 ± 0.6	1.5 ± 0.7	1.5 ± 0.7	1.5 ± 0.8	1.2 ± 0.5
